# Post‐glacial colonization of the Fennoscandian coast by a plant parasitic insect with an unusual life history

**DOI:** 10.1002/ece3.9996

**Published:** 2023-04-18

**Authors:** Christer Solbreck, Anna Cassel‐Lundhagen, Ane T. Laugen, Peter Kaňuch

**Affiliations:** ^1^ Department of Ecology Swedish University of Agricultural Sciences Uppsala Sweden; ^2^ Department of Natural Sciences University of Agder Kristiansand Norway; ^3^ Institute of Forest Ecology Slovak Academy of Sciences Zvolen Slovakia; ^4^ Department of Zoology, Institute of Biology and Ecology P. J. Šafárik University in Košice Slovakia

**Keywords:** aeroplankton, Cecidomyiidae, coalescence time, diapause, genetic structure, individual heterozygosity

## Abstract

Species that exhibit very peculiar ecological traits combined with limited dispersal ability pose a challenge to our understanding of ecological and evolutionary mechanisms. This is especially true when they have managed to spread over long distances, overcome physical barriers, and colonize large areas. Climate and landscape changes, trophic web relations, as well as life history all interact to shape migration routes and present‐day species distributions and their population genetic structures. Here we analyzed the post‐glacial colonization of northern Europe by the gall midge *Contarinia vincetoxici*, which is a monophagous parasite on the perennial herb White swallowwort (*Vincetoxicum hirundinaria*). This insect not only has a narrow feeding niche but also limited dispersal ability and an exceptionally long dormancy. Gall midge larvae (*n* = 329) were collected from 16 sites along its distribution range in Denmark, Sweden, and Finland. Using microsatellite loci and knowledge of the species and the regions' history, we investigated the role of landscape change, host plant distribution, insect population dynamics, and life history in shaping the population genetic structure of the insect. We devoted particular interest to the role of the insect's presumed poor dispersal capacity in combination with its exceptionally extended diapause. We found significant levels of local inbreeding (95% highest posterior density interval = 0.42–0.47), low‐level within‐population heterozygosity (mean *H*_E_ = 0.45, range 0.20–0.61) with private alleles in all populations except two. We also found significant (*p* < .001) regional isolation‐by‐distance patterns, suggesting regularly recurring mainly short‐distance dispersal. According to approximate Bayesian computations, *C. vincetoxici* appears to have colonized the study area via wind‐aided flights from remote areas approximately 4600–700 years before present when the land has gradually risen above the sea level. Extremely long dormancy periods have allowed the species to “disperse in time”, thereby aiding population persistence despite generally low census population sizes.

## Graphical abstract

We analyzed the post‐glacial colonization of northern Europe by an insect with a narrow feeding niche, limited dispersal ability and an exceptionally long dormancy. We found substantial levels of local inbreeding and regional isolation‐by‐distance patterns. The species colonized the study area via wind‐aided flights from remote areas approximately 4600–700 years before present when the land has gradually risen above the sea level.

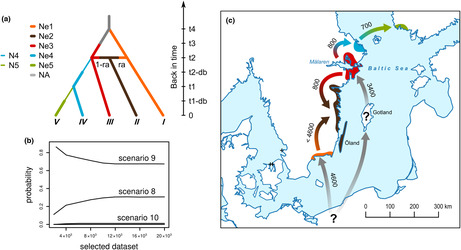

## 1 INTRODUCTION

The northern European flora and fauna is relatively young, having gradually colonized the region since the last glaciation (Hewitt, 1999). The establishment success of colonizing species depends on the presence of suitable living conditions, such as climate, food resources, and habitats. However, the colonization success is also affected by the specific biology and life history of the colonizers; their migratory habits, generation time, and reproductive potential (Mayer et al., 2015). Therefore, whereas some species colonized northern Europe long ago (soon after the ice sheet retreated), other species are later arrivals as they may be limited by poorer dispersal capacity or unsuitable living conditions. Many species are also dependent on prior arrival by other organisms. This is, for example, illustrated by insect species that are confined to a parasitic life inside a single host plant species where the biology and spread of the host plant set the limits for the focal species (Goczal et al., 2020; Mayer et al., 2015).

**FIGURE 1:**
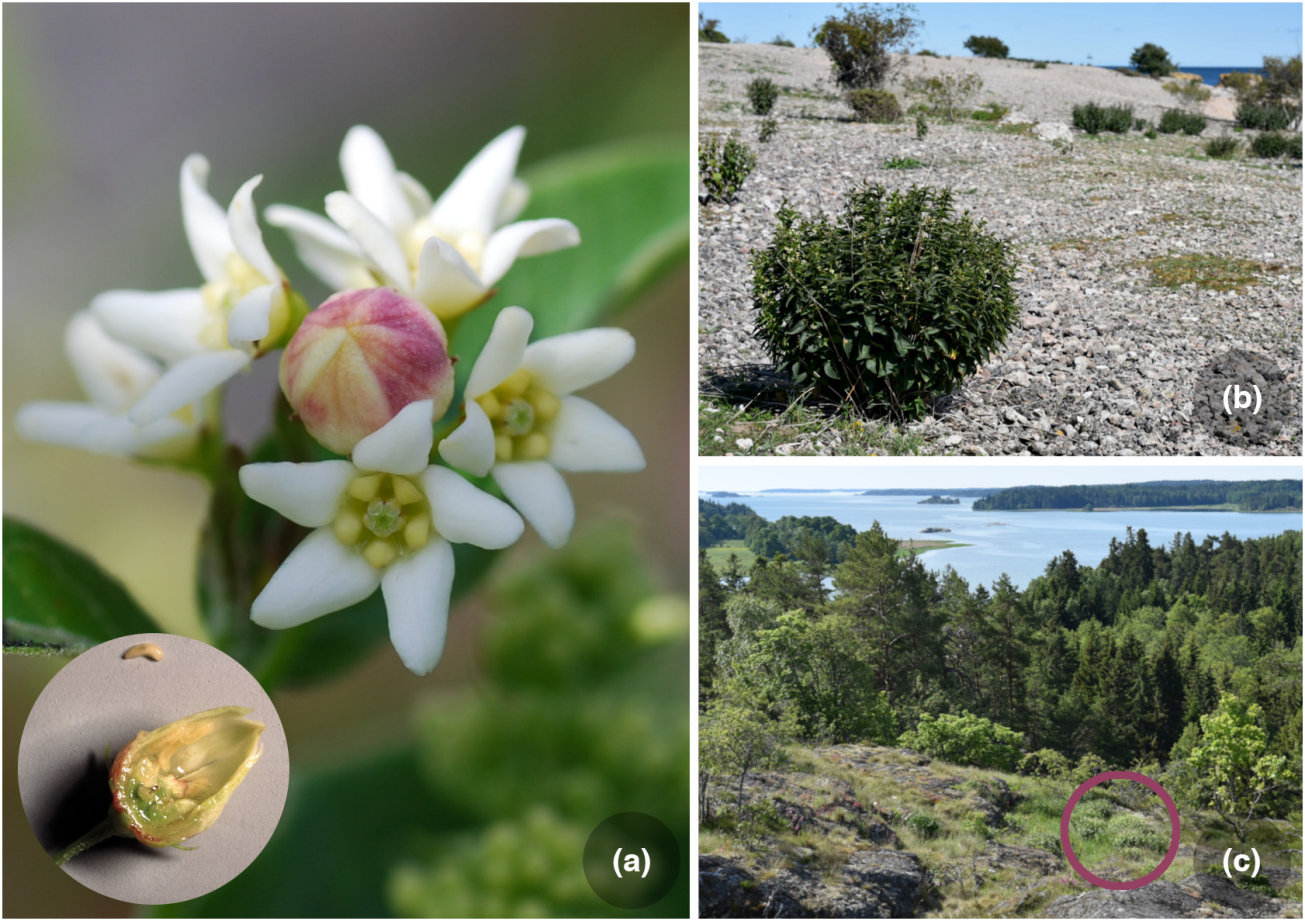
(a) A gall of *Contarinia vincetoxici* midge (Diptera, Cecidomyiidae) on White Swallowwort, *Vincetoxicum hirundinaria* flower with a detail showing larvae clinging to the inner wall of the gall. (b) An individual plant on a beach habitat (early colonizing site) and (c) an old plant patch (circle) on a rock shelf (Photo by Christer Solbreck).

Here we investigate the post‐glacial colonization process of a plant‐parasitic insect *Contarinia vincetoxici* (Diptera, Cecidomyiidae) with an unusual life history. This monophagous gall midge is a tiny insect whose larva lives in flower galls of the perennial herb White Swallowwort, *Vincetoxicum hirundinaria* (Gentianales, Apocynaceae; Widenfalk et al., 2002). The insect's life is characterized by a brief period of active life and growth followed by a very long inactivity period (Solbreck & Widenfalk, 2012). The active period commences when the short‐lived adults (1–2 days) emerge in early summer. They fly and mate soon after emergence, and females then oviposit in young flower buds that are transformed into galls where the larvae develop (Figure 1a). Each gall contains about 15 larvae, and it appears that females can produce 1–2 galls each. The larvae feed for about 2 weeks before they leave the gall to spin a cocoon in the soil (Widenfalk et al., 2002). This is the beginning of the exceptionally long inactivity period spent in diapause (Danks, 1992) with a median duration of at least 6 years (range 2–13 or even more years; Solbreck & Widenfalk, 2012). The many age classes of diapausing gall midge larvae form a dormant population in the soil, which acts as an effective buffer against local extinctions (Solbreck & Widenfalk, 2012, 2020; Widenfalk & Solbreck, 2005). Another important aspect of *C. vincetoxici* life history is its limited dispersal ability. Like other gall midge species (Sylvén, 1970) adults are poor fliers with very limited power to move upwind in search for host plants. Earlier studies of *C. vincetoxici* show that colonization of empty host plant patches is uncommon even at short distances from inhabited patches (Solbreck & Widenfalk, 2020). However, from a postglacial timescale perspective, we cannot rule out long distance and/or wind‐aided flights in *C. vincetoxici*. Other gall midge species are known to enter the aeroplankton and to fly high above the ground (Johnson, 1969; Sylvén, 1970). To summarize, this gall midge is a slow and somewhat unpredictable colonizer but a sturdy survivor.

**FIGURE 2:**
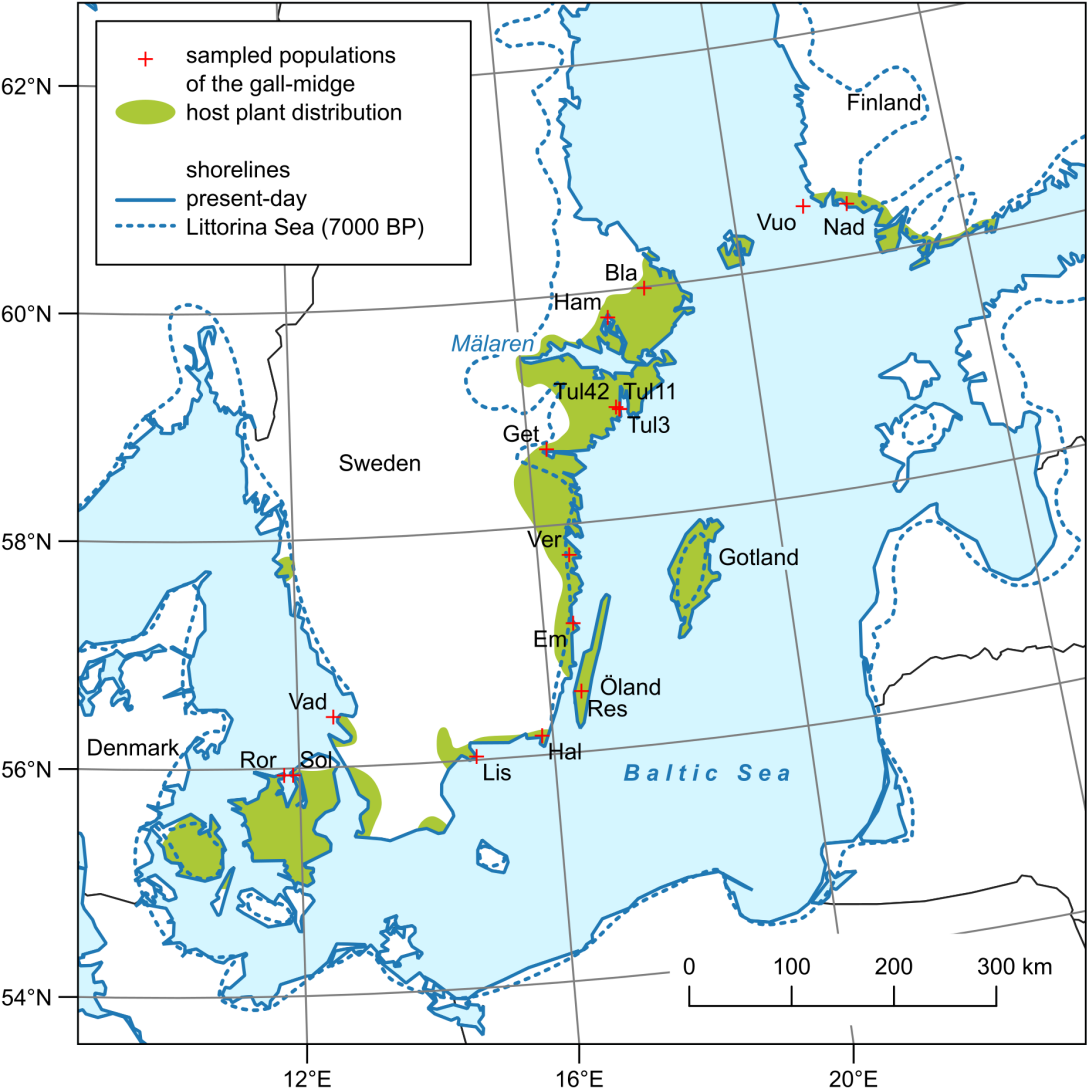
Location of sampled gall midge *Contarinia vincetoxici* populations and the known current distribution area of its host plant *Vincetoxicum hirundinaria* along the Baltic coast in Fennoscandia. Post‐glacial shoreline of the Littorina Sea depicts the area where occurrence of both species was impossible in this historical period.

The long‐lived host plant is found in rocky sun‐exposed habitats (Figure 1b,c). It often has a distinct patchy distribution on the landscape scale (Solbreck, 2012). Its geographical range covers most of Europe and parts of northern Asia with a north‐western distribution border along the south‐eastern coast of Sweden, eastern Denmark, and the south‐western corner of Finland (Donadille, 1965; Hultén & Fries, 1986). The gall midge has a more limited distribution range than its host plant and it is found in northern Italy, western Germany, Czechia, Slovakia, Austria, Switzerland, Belgium, Denmark, Sweden, and Finland (Skuhravá, 1986; Skuhravá et al., 2006; Skuhravá & Skuhravý, 2010; Widenfalk et al., 2002). Two distribution areas of the gall midge can be discerned. First, a central area around the Alpine countries and central Europe, and secondly, an arm extending along the western side of the Baltic Sea in Sweden and Denmark moving into the south‐western tip of mainland Finland (Figure 2).

Along the “Fennoscandian arm” the insect distribution today closely tracks the distribution limit of the host plant, and the insect is found in most places where the host plant grows. However, host plant distribution has evidently changed much during the post‐glacial period. Whereas the plant is believed to have colonized much of the south‐eastern Swedish coast already 7000–8000 BP (Sterner, 1922), its further distribution (from Lake Mälaren region to SW Finland) was initially hindered. Much of the northernmost part of our study area was submerged and has gradually risen above the sea level during the last 7000 years (Harff & Meyer, 2011; Karlsson, 2007). This process has affected plant spread (Muola et al., 2021) with likely effects on the geographic structuring of gall midge populations.

Colonization processes can be detected by analyzing the variation of neutral molecular markers, e.g., microsatellite loci (simple sequence tandem repeats of nuclear DNA), which are routinely and effectively employed for such a purpose. Multilocus genotypes mirror the diversity of founders, dispersal pathways, and divergence times of populations (Austerlitz et al., 1997). Based on the gall midge's unique biology and the distribution pattern of its host plant, we aimed to (1) infer population structure and spatial differentiation of populations of the species and (2) reconstruct their colonization history using a coalescent‐based approximate Bayesian computation (Cornuet et al., 2014). Finally, we aimed to (3) analyze indices of genetic variation that allow conclusions to be drawn about the history of the species and also reveal other biological phenomena (e.g., inbreeding) in the study system. We hypothesized that the insect has colonized the Baltic coast in Fennoscandia through a gradual expansion in a stepping stone‐like manner (Kimura & Weiss, 1964). We expected that the gall midge has colonized new areas through very slow and successive local encounters of new suitable habitat patches that have emerged along the coast as the land rose above the sea level. However, we also expected signs of rare long‐distance colonization events, creating pockets of unique genotype combinations. From the above‐mentioned colonization hypothesis, we derived two predictions regarding genetic variation. First, we predicted a correlation between genetic and geographic distances only at short distances with a breakdown of correlation at larger distances, but with pockets of genetic variants disrupting the general isolation‐by‐distance pattern. Second, we predicted a higher genetic variability in older introductions as a consequence of accumulation of novel mutations.

## 2 MATERIALS AND METHODS

### 2.1 Sampling of gall midge populations

A total of 329 galls were collected from 16 sites ranging from SW Finland to SE and S Sweden and E Denmark (Table 1; Figure 2). For the analysis, one larva was chosen from each gall. Populations were sampled in 2012 or 2015 except for three sites (*Vad*, *Sol*, *Ror*) which were sampled in 1991. This sampling covers the current distribution of the host plant and gall midge populations in Fennoscandia. When galls were few at a site, they were all collected. When they were more numerous, collecting efforts were spread across the entire host plant patch.

**TABLE 1:** Overview of 16 populations of *Contarinia vincetoxici* from the Baltic coast in Fennoscandia sampled mostly in 2012 or 2015.

Site (country)	Code	Latitude N	Longitude E	*n*	*N*_A_	*P*_A_	*H*_O_	*H*_E_	*F*_IS_
Vuosnainen (FI)	Vuo	60°31′	21°15′	4	26	1	0.23	0.25	0.34
Nådendal (FI)	Nad	60°28′	22°01′	22	32	0	0.17	0.20	0.35
Bladåker (SE)	Bla	59°59′	18°16′	28	30	1	0.19	0.25	0.39
Hammarskog (SE)	Ham	59°46′	17°35′	21	48	4	0.32	0.47	0.36
Tullgarn 42 (SE)	Tul42	58°59′	17°33′	30	74	6	0.45	0.57	0.32
Tullgarn 3 (SE)	Tul3	58°58′	17°37′	27	65	1	0.33	0.44	0.37
Tullgarn 11 (SE)	Tul11	58°58′	17°37′	24	60	0	0.39	0.50	0.31
Getå (SE)	Get	58°40′	16°19′	26	70	4	0.42	0.55	0.34
Verkebäck (SE)	Ver	57°44′	16°31′	34	81	3	0.40	0.58	0.37
Em (SE)	Em	57°08′	16°29′	28	77	4	0.51	0.61	0.30
Resmo (SE)	Res	56°32′	16°31′	27	89	14	0.47	0.61	0.33
Hallands Väderö (SE)	Vad	56°26′	12°34′	4	21	2	N/A	0.44	0.35
Hallarum (SE)	Hal	56°10′	15°50′	19	53	2	0.34	0.43	0.31
Lister (SE)	Lis	56°02′	14°47′	24	43	2	0.29	0.43	0.40
Sölager (DK)	Sol	55°56′	11°55′	5	24	1	N/A	0.56	0.60
Rörvig (DK)	Ror	55°56′	11°46′	6	35	1	0.26	0.35	0.35
Mean					52	3	0.34	0.45	0.36

Abbreviations: *F*_IS_, inbreeding coefficient; *H*_E_, expected heterozygosity; *H*_O_, observed heterozygosity; *n*, number of genotyped individuals; *N*_A_, number of alleles; *P*_A_, number of private alleles.

### 2.2 DNA extraction and microsatellites

Developing larvae were dissected out from galls, and DNA was then extracted from one larva per gall using a Qiagen DNeasy Tissue Kit (Qiagen) according to manufacturer instructions. Microsatellite sequences of *C*. *vincetoxici* were isolated by ecogenics GmbH using the high‐throughput genomic sequencing approach described by Abdelkrim et al. (2009). A total of 1 μg of genomic DNA was analyzed on a Roche 454 GS‐FLX platform (Roche) using a 2/16th run and the GS FLX titanium reagents. The total 94,738 reads had an average length of 288 base pairs. Of these, 1761 contained a microsatellite insert with a tetra‐ or a trinucleotide of at least six repeat units or a dinucleotide of at least 10 repeat units. Altogether, 733 reads containing microsatellite inserts were suitable for primer design, 30 of which were randomly selected and tested for polymorphism. Finally, after excluding loci that did not amplify well or were difficult to interpret, the samples were genotyped using a set of 15 polymorphic loci amplified in three multiplex PCR (Appendix S1). The fragment analysis was performed at SciLifeLab Uppsala. Fragment lengths in electropherograms were determined by comparison of the detected true peak signals against the LIZ500 size standard (Applied Biosystems) using PeakScanner (Applied Biosystems) and manual editing. To ensure correct sizing of alleles, we re‐amplified any loci that did not show clear peaks at least once. If some individuals were still missing a peak signal, the locus in question was treated as missing data.

To test for genotypic linkage disequilibria among loci, we used contingency tables and an unbiased estimate of the exact probability obtained by Markov Chain Monte Carlo simulations and 10,000 permutations using Arlequin 3.5 (Excoffier et al., 2005). We also checked microsatellite loci for Hardy–Weinberg equilibrium (HWE) and frequency of null alleles was estimated using methods of Chakraborty et al. (1994) and Brookfield et al. (1996) implemented in the “PopGenReport” 3.0.4 package (Adamack & Gruber, 2014) of the R 3.6.3 software (R Core Team, 2020). HWE was tested for each combination of sampling site and locus by the Chi‐square test with the Yates continuity correction and with Bonferroni adjustment to prevent type I errors (α = 0.05/240). To avoid biased estimates of null alleles, we used the INEst 2.2 software (Chybicki & Burczyk, 2009) for simultaneous estimation of null allele frequencies and of the inbreeding coefficient to avoid biased estimates of null alleles.

### 2.3 Genetic diversity and distances

Genetic diversity indices (number of alleles, number of private alleles, observed and expected heterozygosity) and basic *F* statistics were calculated using the R packages “adegenet” 2.1.3 (Jombart, 2008) and “hierfstat” 0.5‐8 (Goudet, 2005), respectively. We initially explored genetic relationship between populations using an unrooted neighbor‐joining tree based on shared allele distances (maximum‐likelihood with 1000 bootstrap replicates), calculated in the software Populations 1.2.33 (Langella, 1999) and constructed in the R package “ape” 5.3 (Paradis & Schliep, 2019). To test for isolation by distance (IBD) in sampled populations, we used the Mantel test between matrices of genetic and geographic distances with 999 permutations in the R package “adegenet”. For the genetic matrix we used chord distance *D*_C_ (Cavalli‐Sforza & Edwards, 1967), which is the most powerful estimate for datasets with possible null alleles (Séré et al., 2017).

### 2.4 Population structure analysis

To find genetically homogeneous groups of individuals in our samples, we used an individual‐based clustering method implemented in the software Structure 2.3.4 (Hubisz et al., 2009; Pritchard et al., 2000). We ran the admixture model with correlated allele frequencies without the prior population information and degree of admixture α = 1. For each value of *K* (range 1–16), we conducted 20 independent runs with uniform priors using a burn‐in of 100,000 iterations followed by 100,000 Markov chain Monte Carlo iterations. The number of genetic clusters *K* in the data set was inferred in two steps. Firstly we used the Δ*K* method (Evanno et al., 2005), which finds the breakpoint in the slope of the likelihood distribution for different *K* values, using the Structure Harvester Web 0.6.94 (Earl & von Holdt, 2012). Thereafter, we identified the stable *K* solutions also through Q‐matrix correlations (average maximum correlation coefficient and the rows‐and‐columns method) implemented in the R package “CorrSieve” 1.6‐9 (Campana et al., 2011), which helps to identify anomalous runs. In our structure analysis, we adopted the hierarchical approach (Evanno et al., 2005). Thus, the uppermost hierarchical level of detected optimal *K* was also analyzed independently to detect potential substructure within inferred main clusters (*K* ranged from 1 to a total number of sampling sites included in the analyzed cluster). Outputs of the Structure analysis were visualized with the Clumpak program (Kopelman et al., 2015).

To compare variability among determined genetically homogenous clusters, we used four indices of individual heterozygosity, which are relevant for populations with high inbreeding (Aparicio et al., 2006): (1) proportion of heterozygous loci (*PHt*), (2) standardized heterozygosity based on the mean expected heterozygosity (*Hs_exp*), (3) internal relatedness (*IR*), and (4) homozygosity by locus (*HL*) calculated by the R function GENHET (Coulon, 2010). We determined differences among groups by the nonparametric Kruskal–Wallis ANOVA with a post hoc Dunn test for multiple comparisons in the R package “FSA” 0.8.30 (Ogle et al., 2020).

### 2.5 Inferring colonization scenario

To estimate divergence times and population history of gall midge populations, we used a coalescent‐based Approximate Bayesian Computation (ABC) algorithm in the program DIYABC 2.1.0 (Cornuet et al., 2014). We assigned the sampling sites to genetic clusters identified by the Structure analysis. The scenarios of the compared coalescent models, which we defined as the most likely, were based on the following three assumptions or hypotheses. Our first assumption was that colonization of the area was gradual (stepping stone model; Kimura & Weiss, 1964) as the land was uncovered after the sea level retreated, thus, the species progressed from south to north (from cluster *I* to *V*; scenario 1 of Figure S1). The second assumption was that colonization began in the area where the highest diversity was found (cluster *II* or clusters *II* + *III*), indicating a long‐term effect of mutations in a large ancestral population (scenarios 2–6). Lastly, we added a competing hypothesis where the population with the highest variation resulted from the admixture of different independently introduced lineages from an unknown source that gradually colonized the territory from different directions (scenarios 7–10). For the second and third assumptions, there are several alternative scenarios that differ in the times of divergence or admixture of different clusters. From these hypotheses, we selected a set of 10 hypothetical or possible evolutionary scenarios (Figure S1), which were designed based on the results of the Structure analysis, and we simulated demographic parameters for inferred genetic clusters. Since the DIYABC software is strongly influenced by differences in sample size between populations, we selected 25 individuals for each cluster using random sub‐sampling with respect to the smallest cluster. We explored the coalescence time (in generations and where a median generation time was estimated to 6 years based on field observations, Solbreck & Widenfalk, 2012) since population divergence, the time and rate of population admixture and discrete change in effective population size. We used a default uniform distribution and priors for all parameters. Because effective population sizes (Ne) in sampled sites were calculated very low (<100) using the linkage disequilibrium method as implemented in NeEstimator 2.1 (Do et al., 2014), Ne was set in a range from 10 to 1500 which corresponds better to our field estimates. An initial size reduction (bottleneck) is expected when a new populaton is derived from an ancestral population, since this new population generally starts with few founders – a typical feature of the study system. Therefore, we set also an initial size reduction in different scenarios with the number of founders (N) in a range from 1 to 100. Priors for divergence times (in generations) were set as follows: t1 = 10–500, t2 = 100–500, t3 = 100–1000, t4 and t5 = 500–3000; while t5 > t4, t5 > t3, t5 > t2, t5 > t1, t4 > t3, t4 > t2, t4 > t1, t3 > t2, t3 > t1 and t2 > t1. An admixture rate (ra) ranged from 0.001 to 0.999. An equal prior probability was assumed for each competing scenario. For 15 microsatellite loci (number of alleles ranged from 8 to 18), we set a default range of 40 contiguous allelic states as suggested (Cornuet et al., 2014) and assumed a Stepwise Mutation model. The mean mutation rates (μ) of loci were set to follow a uniform distribution with the prior rate per locus per generation in a range from 10^−4^ to 10^−3^ with individual loci variation from 10^−5^ to 10^−2^ following Gamma distribution. The mean geometric distribution parameter (P) was set to 0.22. We generated a reference table containing 10 million datasets, each scenario being given a uniform prior probability. Posterior probabilities of each scenario were estimated using local logistic regression on 1% of simulated datasets closest to the observed dataset after applying a logit (default) transformation of demographic and historical parameter values. Three populations (*Vad*, *Ror*, *Sol*) with the smallest sample size and low genotyping success were not used in this ABC analysis.

## 3 RESULTS

### 3.1 Genetic variation

We successfully genotyped 329 larvae of *C. vincetoxici* (4–34 individuals per population) using 15 microsatellite loci. In total, 3.2% of data was missing. However, this was particularly due to the three populations with the smallest sample size (*Vad*, *Ror*, *Sol*) where on average 5 out of 15 loci were not amplified. Significant linkage disequilibria were found for almost all loci combinations (Table S1). However, there was no consistency among populations, and no locus stood out more than another, thus we interpret the pattern as being a result of a biological phenomenon rather than a pattern created by physical linkage among loci. Significant departures (*p* < .001) from HWE were revealed in half of the populations at 1–6 loci (Table S2). Although there was also evidence of null alleles at all loci (Table S3), their adjusted frequencies by simultaneous estimation of the inbreeding coefficient indicated relatively low values (up to 10%) of null alleles at different loci (Table S3). However, because deviations from HWE between loci in different populations were completely random (Table S2), we left the dataset as is for further analyses and did not compensate for null alleles.

Used 15 loci revealed relatively high variability in a number of alleles in different populations (*N*_A_ = 21–89) but showed very low within‐population observed (*H*_O_ = 0.17–0.51) and expected heterozygosity (*H*_E_ = 0.20–0.61) per population (Table 1). Unique (private) alleles were found in all populations except two (*Nad*, *Tul11*). The exceptionally high number of private alleles (14 out of 89 alleles) was found in the population *Res* on Öland Island (Table 1). Population inbreeding *F*_IS_ ranged from 0.30 to 0.60 (Table 1). However, posterior mean inbreeding coefficient for the whole dataset was 0.45 (95% highest posterior density interval was 0.42–0.47) and provided strong support for a presence of very high inbreeding in the analyzed populations. The deviance information criterion (DIC) confirmed that the full model including inbreeding coefficients outperformed a model that had only null alleles and genotyping failures involved.

### 3.2 Population genetic structure

**FIGURE 3:**
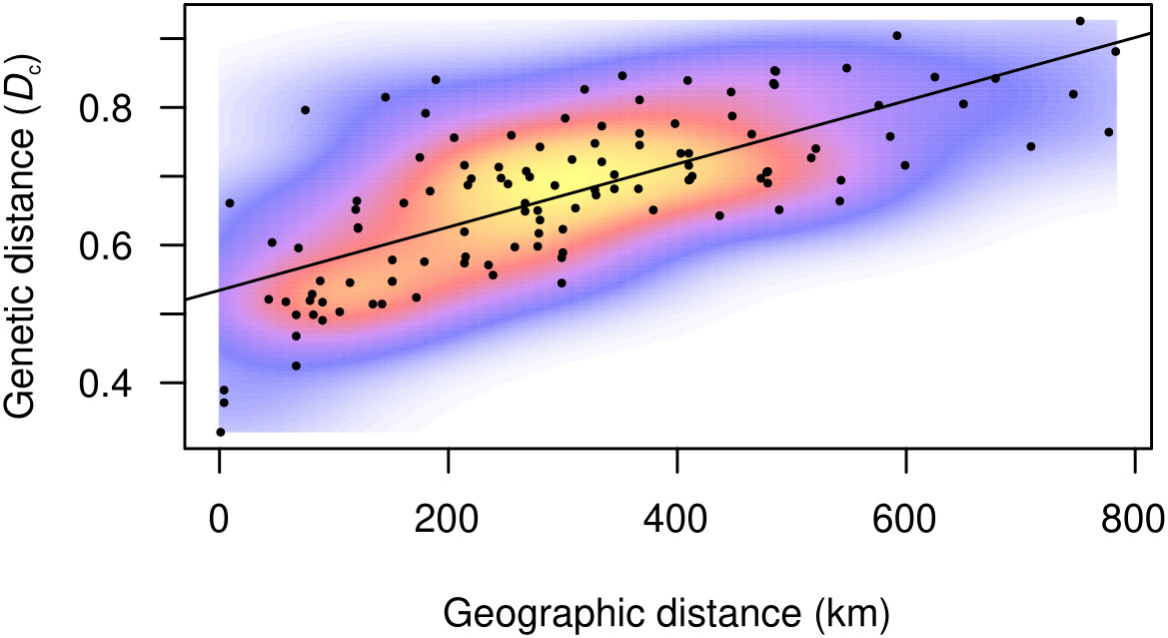
Isolation by distance scatterplot of *Contarinia vincetoxici* populations in Fennoscandia.

**FIGURE 4:**
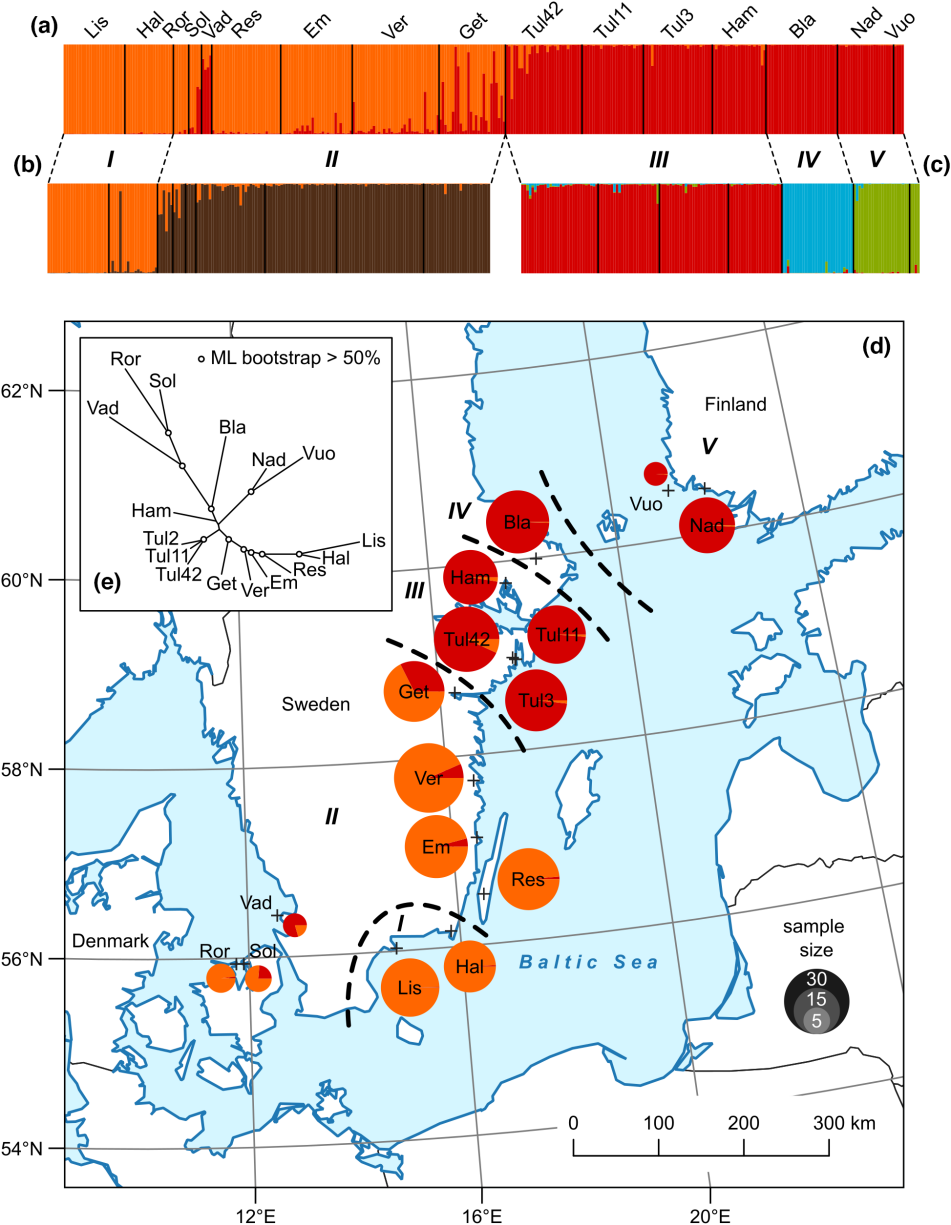
Genetic structure of *Contarinia vincetoxici* samples in Fennoscandia as inferred by the Structure simulations using a hierarchical clustering approach (final clusters labeled *I*–*V*). (a) Full dataset, which identified two main clusters; (b) samples within southern subset of populations; (c) samples within northern subset of populations. Individuals in the bar plots are represented by vertical bars divided into parts proportional to their proposed ancestry in each genetic cluster. (d) Ancestry proportions in samples (crosses along the coast) according to uppermost hierarchical level of clustering. (e) An unrooted neighbor‐joining tree based on shared allele distances.

**FIGURE 5:**
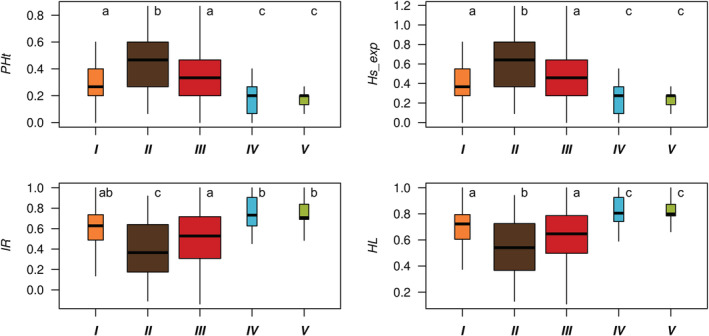
Indices of individual heterozygosity of *Contarinia vincetoxici* (*PHt*, proportion of heterozygous loci; *Hs_exp*, standardized heterozygosity; *IR*, internal relatedness; *HL*, homozygosity by locus) in five genetic clusters (*I*–*V*). Width of boxes is relative to the sample size. Different lower‐case letters denote groups that significantly differ (*p* < .05) from the others according to post hoc Dunn tests.

Overall measures of genetic variation, both within‐ and among‐population components, quantified with Wright's fixation indices were as follows: *F*_ST_ = 0.33 (pop/total), *F*_IT_ = 0.51 (ind/total) and *F*_IS_ = 0.27 (ind/pop). Contrary to our prediction, the IBD analysis resulted in a continuous cline of genetic differentiation (Mantel test; *R* = .72, *p* < .001) with no sign of pattern breakdown (Figure 3, Figure S2). Both methods, Δ*K* and Q‐matrix correlations, detected a stable genetic structure at *K* = 2 when the full dataset was analyzed (Figure 4a), but the hierarchical approach inferred further structuring within both the southern (clusters *I* and *II*; Figure 4b) and the northern subsets. Although the Evanno method indicated four more clusters in the northern populations, inconsistency between the maximum values of Δ*K* and *F*_ST_ according to the number of clusters suggested an unstable resolution (Figure S3). Since the maximum average Q‐matrix correlation also yielded a stable solution for *K* = 3 (*R* = .99, *p* < .05), we therefore assumed two genetic clusters around the lake Mälaren in central Sweden (clusters *III* and *IV*) and one cluster of populations from southern Finland (cluster *V*; Figure 4c). Finally, we found five genetic clusters, derived from two main clusters, which followed a latitudinal pattern along the Baltic Sea coast (Figure 4d). Three populations located in SW Sweden (Hallands Väderö) and E Denmark (Sölager and Rörvig) did deviate from this pattern, but their relative genetic distance from other samples (Figure 4e) was likely biased by small sample sizes and lowered genotyping success. The pattern was also stable when the structure analysis was rerun by a reduced number of 10 loci where the loci that did not amplify well were omitted (data not shown). In general, there was no difference in the level of inbreeding between genetic clusters (Figure S4), but individual heterozygosity differed for all indices (*PHt*, χ^2^ = 67.7, df = 4, *p* < .001; *Hs_exp*, χ^2^ = 67.7, df = 4, *p* < .001; *IR*, χ^2^ = 59.8, df = 4, *p* < .001; *HL*, χ^2^ = 73.8, df = 4, *p* < .001). This analysis suggested the highest heterozygosity in cluster *II* (Figure 5).

### 3.3 Colonization history

**FIGURE 6:**
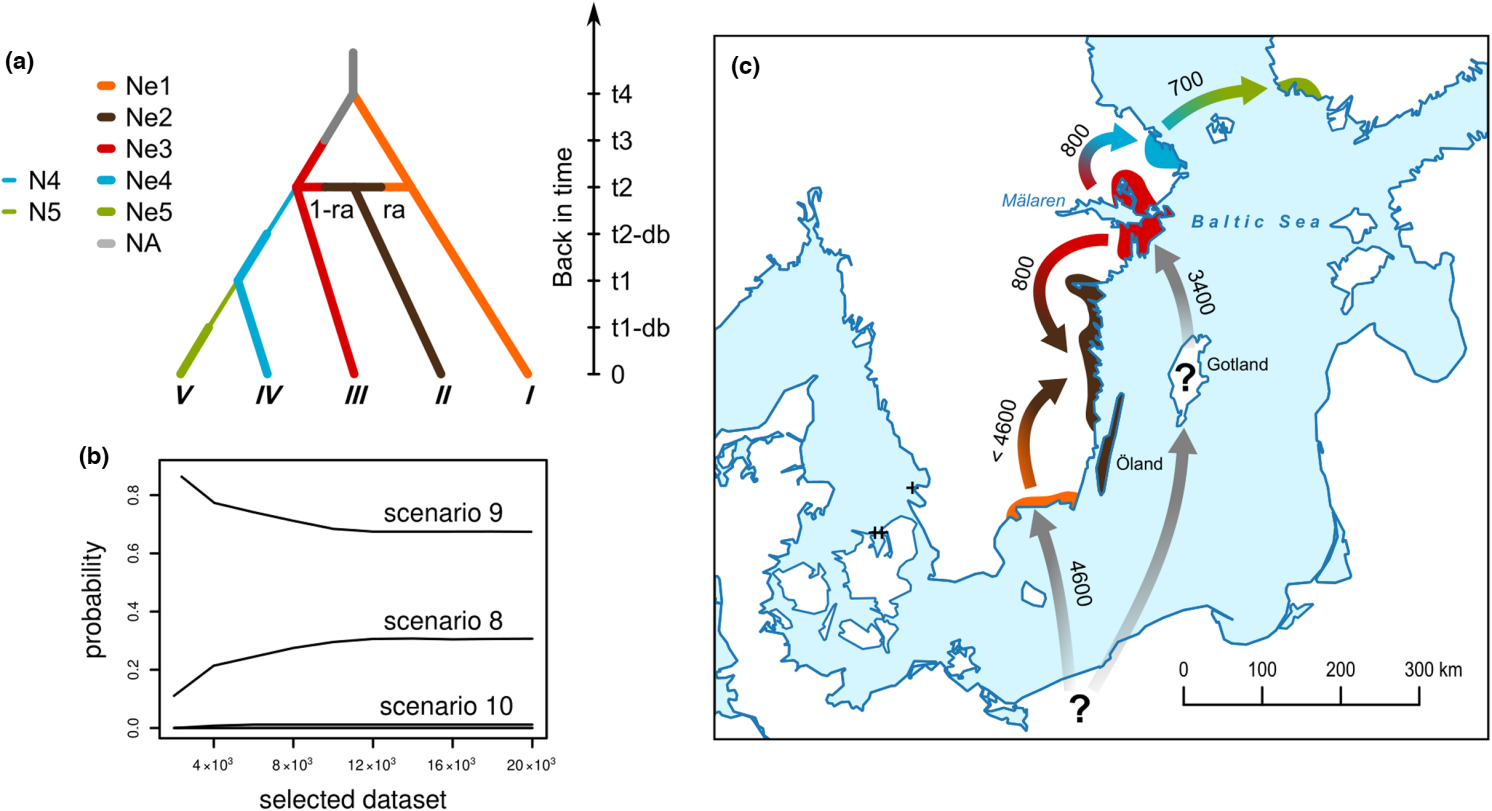
(a) Schematic representation of the best‐supported colonization scenario for *Contarinia vincetoxici* (scenario 9; competing scenarios see in Figure S1) inferred by the Approximate Bayesian Computation analysis (Ne, effective population size; N, initial size reduction; NA, unsampled founder population; t, time of divergence or admixture; db, demographic bottleneck; ra, admixture rate; for simulated parameters and estimated values see Section 2 and 3, respectively). (b) The comparative posterior probabilities of each scenario fitting the real data using logistic regression (please note that other scenarios overlap at the bottom of the plot). (c) Reconstruction of colonization history of the gall‐midge along the Baltic coast in Fennoscandia founded by unknown source populations. Values show approximate years before present (crosses, samples not analyzed; see Section 2).

From the 10 tested possible evolutionary scenarios (Figure S1), scenario 9 (Figure 6a) emerged as the most likely scenario of the Baltic coast colonization by *C. vincetoxici* as deduced from the ABC computations. Posterior probability of this scenario was 0.67 after logistic regression on the 1% simulated data most similar to the observed data; however, probability of scenario 8 (Figure S1) was also relatively high (Figure 6b). The probability with which scenario 8 was rejected although it was the true scenario was 0.32 (type I error; 500 test data sets simulated under selected scenario), and the probability of supporting this scenario even when it was not the correct one was 0.39 (type II error). Median (2.5%–97.5% quantiles) time estimated in numbers of generations since divergence from an unsampled founder population (*N*_A_), outside the study area (or independent introductions of the species to Fennoscandia), was = 768 (516–1960) for the cluster *I* populations (t4) and 566 (210–952) for the cluster *III* populations (t3). Simultaneous admixture of cluster *I* and *III* populations and divergence between cluster *III* and *IV* (t2) was estimated around 133 (103–244) generations ago. Finally, population cluster *V* diverged from cluster *IV* 121 (43–246) generations before present (t1). This is in accordance with our prediction that older populations have higher genetic variability than younger (*I* and *III* vs. *IV* and *V*). However, the highest level of variation was found in the population that ABC analysis suggested as an admixture of two lineages (cluster *II*; Figures 5 and 6). Effective population sizes (median and 2.5%–97.5% quantiles) for five genetic clusters under this scenario were for Ne1 = 322 (79–962), Ne2 = 1410 (1220–1490), Ne3 = 844 (377–1340), Ne4 = 136 (19–816), and Ne5 = 451 (61–1310). If assuming 6 years as the median generation time, the estimated colonization time of *C. vincetoxici* to the study area was approximately 4600–700 years before present (Figure 6c).

## 4 DISCUSSION

The distribution of an organism is the result of intertwined processes operating on a variety of spatial and temporal scales. Our study links the current distribution of the insect to land surface changes, host plant dispersal, and insect life history to draw conclusions on the insect's post‐glacial colonization process. This multiangle approach is essential for understanding colonization routes and present‐day genetic structure of the gall midge *C. vincetoxici* in Fennoscandia. We found relatively low genetic diversity and a high level of local inbreeding, most likely due to the insect's poor dispersal capacity, as evidenced also by a significant isolation‐by‐distance pattern. The presented results also suggest that the species colonized the study area via wind‐assisted flights from remote areas as the land has gradually risen above the level of post‐glacial Littorina sea.

### 4.1 Dispersal capacity

The most striking result from our study is the apparent isolation‐by‐distance (IBD) pattern across the whole study area. Despite the insect being tiny (wing length ~1.5 mm; Widenfalk et al., 2002), it is apparently capable of dispersal that counteracts genetic drift even at distances up to 800 km. Further, we could not see any sign of the expected combination of very short dispersal with rare long‐distance expansions. Rather, it appears that colonization of the northern distribution area has relied on a gradual and relatively strong dispersal capacity. A hierarchical population structure can easily be mistaken for a strong pattern of IBD, but the reverse is also true (Meirnmans, 2012). However, knowledge of colonization history can disentangle the problem. The uppermost hierarchical level in our approach inferred only two main clusters. This is in accordance with two independent introductions that appears to have occurred approximately 300 km apart, as detected by ABC analysis (~56°N vs. 58°N). Since further genetic clusters were inferred only in the substructures at shorter geographical distances we hypothesize that the presented genetic structure is due to colonization events rather than a consequence of IBD (Perez et al., 2018).

So how does this insect traverse the landscape? Observations on *C. vincetoxici* and of gall midges in general agree with the picture of a mixed dispersal strategy. A slow short‐distance dispersal seems to be the dominating colonization process when looked at in the moderate time perspective of the population ecologist. Expansions to previously unoccupied patches are uncommon and are usually confined to rather short distances from previously colonized patches (Solbreck & Widenfalk, 2020). However, occasional long‐distance dispersal events in gall midge species seem to appear via aerial plankton (Johnson, 1969; Sylvén, 1970). Even though these long‐distance movements may be too rare to be observed directly, they are obviously important in the long‐time perspective, for example, for an initial colonization of the new area from remote source population. In our case, this could be the transport of founder individuals from the mainland to Fennoscandia. We know that long distance aerial dispersal occurs in many small organisms. For instance, recently ice‐free lands after retreat of glaciers are colonized by aeroplankton of nematodes, mites, spiders, springtails, tardigrades, thrips, and other small insects (Ficetola et al., 2021) enabling gene flow between populations in very remote sites (Ptatscheck et al., 2018).

Wind‐aided flights of adult gall midges provide fast long‐distance transport. For example, a few hours are enough for a flight north‐westwards from Gotland toward the Swedish mainland under suitable weather conditions (C. Solbreck, unpublished data). Furthermore, being part of the natural life cycle, flight is timed to occur when host plants are suitable for oviposition, thus facilitating colonization. Other means of long‐distance dispersal are much less plausible in the gall midge. Zoochory is unlikely as the host plant is poisonous (Kalske et al., 2014; Tullberg et al., 2000) and it is not fed upon by any vertebrates. Drift with plant material on the sea surface (Mende et al., 2010) is slow and hazardous, and it is highly unlikely to provide the synchrony between insect and plant life cycles necessary for successful colonization. Finally, unintentional long transport of species by humans, especially plants with galls containing larva, is also very unlikely, as larvae readily leave disturbed galls.

### 4.2 Colonization events

Our data indicate that long‐distance dispersal should have occurred in this species. This is interesting from an evolutionary perspective as it is probably the way in which *C. vintetoxici* first arrived to the south of the Scandinavian Peninsula in 4.6 ka BP (cluster *I*). Revealed molecular dating also rejects the possibility that dispersal occurred through the last land bridge which connected south tip of the peninsula with the mainland yet 10.3 ka BP before formation of the Littorina/Baltic Sea (Björck, 1995). Another interesting step in the species history is the second introduction event at approximately 3.4 ka BP (cluster *III*). Two pieces of evidence suggest that this may have occurred through aerial dispersal from the island of Gotland. First, there are no records of the gall midge along the eastern Baltic, and secondly, there are large populations of the host plant and numerous observations of the gall midge on this island (C. Solbreck, unpublished data). Unfortunately, we have no genetic data from Gotland and we cannot rule out the possibility of wind transport from even more distant areas. Based on the abundant occurrence of the host plant, the high genetic diversity of the gall‐midge on the island of Öland, and the fact that most of the southern regions have been above sea level for a long time, we assume that the area of cluster *II* was colonized soon after the first introduction from the cluster *I*, until it mixed with cluster *III* 800 BP.

### 4.3 Life history and population genetics

The prolonged larval diapause (of variable length) is an important aspect of the gall midge's life cycle, with considerable implications for its occurrence and population genetics. As our data show, the limited dispersal rate of the gall midge is not a hindrance to high occupancy in some sites. It appears that the high survival through long dormancy periods acts as a strong buffer against local extinction even in very small gall midge populations (Solbreck & Widenfalk, 2012, 2020). Thus, field observations suggest that even though colonization rates are low, once a local habitat patch is colonized it is highly likely to remain so. Population dispersal and establishment is dependent on the relative rates of colonization and of local survival and indicates that this so‐called poor colonizer becomes an efficient survivor in the long run as “migration in time” compensates for limited migration in space.

Another aspect of the “low colonization—high survival” syndrome is the usually small size of host plant patches. This means that most local gall midge populations are also small, and small populations are generally subjected to high extinction risks. However, only a small fraction of a local population emerges from diapause to reproduce each year thus being exposed to extinction hazards (Solbreck & Widenfalk, 2012, 2020), whereas the larvae remaining in diapause in the soil are sheltered. Any genetic variation is likely to be retained longer in this gene bank of diapausing larvae, which was confirmed in our study. As predicted, the local genetic variation was higher in older populations than in recent colonizations. However, the highest variation and the lowest inbreeding were found in the admixture (cluster *II*) of two different introductions to Fennoscandia, which coincided with subsequent colonization events towards north. In addition, extreme inbreeding was manifested in all local populations by elevated levels of homozygosity and a random pattern of linkage among loci. The fact that linkage included most combinations of loci across all data suggests that—rather than being a technical issue—the phenomenon decided by the species life history. The inbreeding pattern appears to be a result of high incidence of mating between closely related individuals rather than a consequence of geographic isolation of certain sites. On the other hand, lower level of inbreeding and high genetic variation, indicating a large effective population size of the gall‐midge found in the Resmo population on the island of Öland, may be associated with the one of the largest populations of White swallowwort in the region.

### 4.4 Conclusion

Summarizing the large‐scale pattern, the colonization and expansion of *C. vincetoxici* in Fennoscandia appear to have happened more than once and in different time periods following the Littorina Sea maximum. Available host plants have created an open road for the insect to travel along the south‐eastern Swedish coast up to 58–59°N, where there used to be a vast water barrier. This barrier gradually became weaker allowing dispersal of the plant and subsequently of the gall midge. Today, the distribution of this insect tracks the entire natural distribution area of the host plant in Fennoscandia, and it is likely a better colonizer than expected. Long‐distance movements by wind undoubtedly occur, but how common are they? Colonization success results from a balance between rates of arrival and subsequent survival in local host plant patches. The very long diapause provides a mechanism by which local survival rates are relatively high. Thus, rare dispersal events seem to be boosted by this phenomenon. However, the peculiar excess of homozygotes, indicating recurring inbreeding, occurs despite migration in time which compensates for limited migration in space.

## AUTHOR CONTRIBUTIONS

**Christer Solbreck:** Conceptualization (equal); data curation (lead); investigation (lead); writing – original draft (equal); writing – review and editing (equal). **Anna Cassel‐Lundhagen:** Conceptualization (equal); investigation (supporting); writing – original draft (equal); writing – review and editing (equal). **Ane T. Laugen:** Conceptualization (equal); writing – review and editing (equal). **Peter Kaňuch:** Formal analysis (lead); visualization (lead); writing – original draft (equal); writing – review and editing (equal).

## CONFLICT OF INTEREST STATEMENT

None declared.

## Supporting information


Appendix S1

## Data Availability

Microsatellite genotypes are available from the Dryad Digital Repository: https://doi.org/10.5061/dryad.63xsj3v70.
