# Supplementary material for: Post‐glacial colonization of the Fennoscandian coast by a plant parasitic insect with an unusual life history
Source: Ecol Evol. 2023 Apr 18;13(4):e9996. doi: 10.1002/ece3.9996 (PMC10111174; doi:10.1002/ece3.9996)
Supplement: Supplementary file 1 — Appendix S1 [file ECE3-13-e9996-s001.pdf]

Supporting information

Post-glacial colonization of the Fennoscandian coast by a plant parasitic insect with an unusual life history

Christer Solbreck, Anna Cassel-Lundhagen, Ane T. Laugen, Peter Kaňuch

Appendix 1. Overview of microsatellite DNA markers and protocols of multiplex PCR amplification of 15 polymorphic loci of *Contarinia vincetoxici* isolated by ecogenics GmbH (Loci are labeled by locus ID in Tables S1, S2, S3)

locus name	locus ID	primer sequence 5'- 3'	repeat motif	dye
**multiplex 1**				
Convin_02370	1	F: AGAGACGAAGTAACCAGCTC<br>R: CGTCGCTTATTTCTGTTCCTTTC	$(GA)_{16}$	6-FAM
Convin_01805	2	F: TCGCGAAATCAAGTCGCATC<br>R: CTTGCCGGCGCAGAATCG	$(AG)_{15}$	Atto565
Convin_08122	3	F: AGACTCAGCGATGGAGATAAAAG<br>R: AGTGTGATGAATAGTGACAACCG	$(AG)_{16}$	Atto550
Convin_49091	4	F: GCAGGCGAAACTTAAGACGG<br>R: TGGCATAAAACAACCCGCTG	$(GT)_{24}$	Atto532
Convin_63351	5	F: GCTTGGCAAAATCGAACAGC<br>R: GTTGGTCTCGGCAACATACG	$(GA)_{17}$	6-FAM
**multiplex 2**				
Convin_28594	6	F: TCGCCATACAGTTTGATTCACG<br>R: GCGCCAACGACTATTCGATAC	$(AC)_{17}$	Atto532
Convin_74425	7	F: CTTGGCAATTCACTCTGCTG<br>R: TTATATGCCCGGTCGTGTCG	$(AG)_{22}$	Atto550
Convin_58384	8	F: GGGCCGACTGCAAATAAATCC<br>R: AGGAGTAAGACACGACGATTG	$(AG)_{21}$	Atto565
Convin_80324	9	F: TGCATTCGCATCTATGTGTACC<br>R: TGAAGAAGACCCAGAAATTTTAACG	$(TG)_{17}$	6-FAM
Convin_25788	10	F: AGCAGTGTGTGCGTGTATTC<br>R: GCACGGTGTATGAGCAAGAC	$(GT)_{21}$	Atto532
**multiplex 3**				
Convin_73025	11	F: CTCTGTCTGCAAGATGACCAC<br>R: TGCAATCGCTGGCATTCG	$(GT)_{15}$	Atto550
Convin_70939	12	F: TTTGCTTGCATGCCTTGTTC<br>R: TGTGGTATTTATGCACTGACGAC	$(AG)_{16}$	Atto532
Convin_53403	13	F: TTACGGCCGATGGAGATGAG<br>R: AGGCAGAAAGCATACCAACG	$(GA)_{18}$	6-FAM
Convin_64790	14	F: TACAGACTCGCGTTGAGAGG<br>R: GTTGTTGGCGGAAAATGTCG	$(TG)_{18}$	Atto565
Convin_72549	15	F: CCTCGAATATGTGCGTTGCC<br>R: CATTCTCGACATAGCAGTCGC	$(GT)_{16}$	Atto550

PCR Protocol Multiplex 1

Multiplex PCR amplification was optimized to be performed in a 10 µl reaction volume containing 5 µl HotstarTaq master mix (Qiagen, Cat. No 203445), double distilled water, and 0.3 µM of forward and reverse primers each (Table 1). We used the following thermotreatment on a TC-412 Programmable Thermal Controller (Techne): 35 cycles with 94°C for 30 seconds, 56°C for 90 seconds, and 72°C for 60 seconds. Before the first cycle, a prolonged denaturation step (95°C for 15 min) was included and the last cycle was followed by a 30 min extension at 72°C.

**Component**	**Primer mix concentration**	**Amount [µl]**	**Final amount/ concentration**
ddH_2O	-	1.2	1.2 µl
Qiagen 2x HotstarTaq Mastermix [§]	-	5	1 x
Convin_02370 (FAM)	20 µM	0.3	0.3 µM each
Convin_01805 (Atto565)	20 µM	0.3	0.3 µM each
Convin_08122 (Atto550)	20 µM	0.3	0.3 µM each
Convin_49091 (Atto532)	20 µM	0.3	0.3 µM each
Convin_63351 (FAM)	20 µM	0.3	0.3 µM each
DNA	-	2	-

§ Provides a final concentration of 0.5 units HotStarTaq DNA polymerase, 1x PCR buffer with 1.5 mM $MgCl_2$ and 200 µM of each dNTP.

Fragment length distribution Multiplex 1

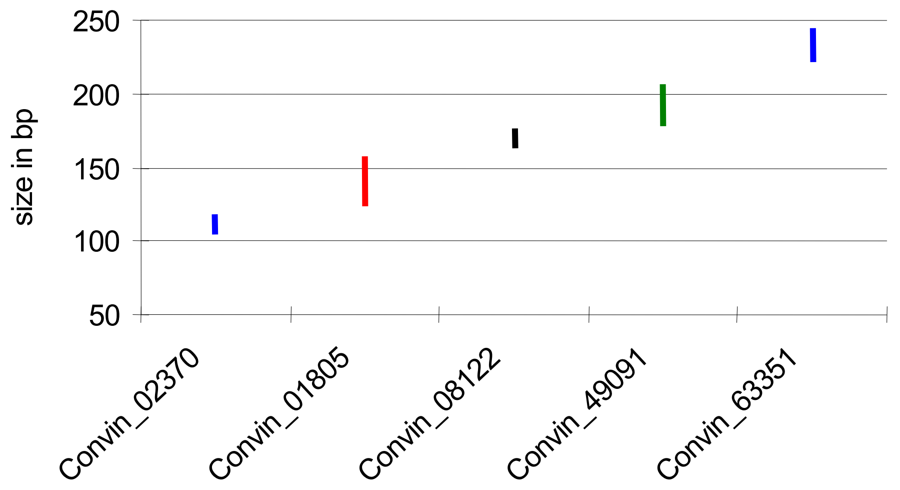

PCR Protocol Multiplex 2

Multiplex PCR amplification was optimized to be performed in a 10 µl reaction volume containing 5 µl HotstarTaq master mix (Qiagen, Cat. No 203445), double distilled water, and 0.3 µM of forward and reverse primers each (Table 3). We used the following thermotreatment on a TC-412 Programmable Thermal Controller (Techne): 35 cycles with 94°C for 30 seconds, 56°C for 90 seconds, and 72°C for 60 seconds. Before the first cycle, a prolonged denaturation step (95°C for 15 min) was included and the last cycle was followed by a 30 min extension at 72°C.

Component	Primer mix concentration	Amount [µl]	Final amount/ concentration
ddH_2O	-	1.2	1.2 µl
Qiagen 2x HotstarTaq Mastermix §	-	5	1 x
Convin_28594 (Atto532)	20 µM	0.3	0.3 µM each
Convin_74425 (Atto550)	20 µM	0.3	0.3 µM each
Convin_58384 (Atto565)	20 µM	0.3	0.3 µM each
Convin_80324 (FAM)	20 µM	0.3	0.3 µM each
Convin_25788 (Atto532)	20 µM	0.3	0.3 µM each
DNA	-	2	-

§ Provides a final concentration of 0.5 units HotStarTaq DNA polymerase, 1x PCR buffer with 1.5 mM $MgCl_2$ and 200 µM of each dNTP.

Fragment length distribution Multiplex 2

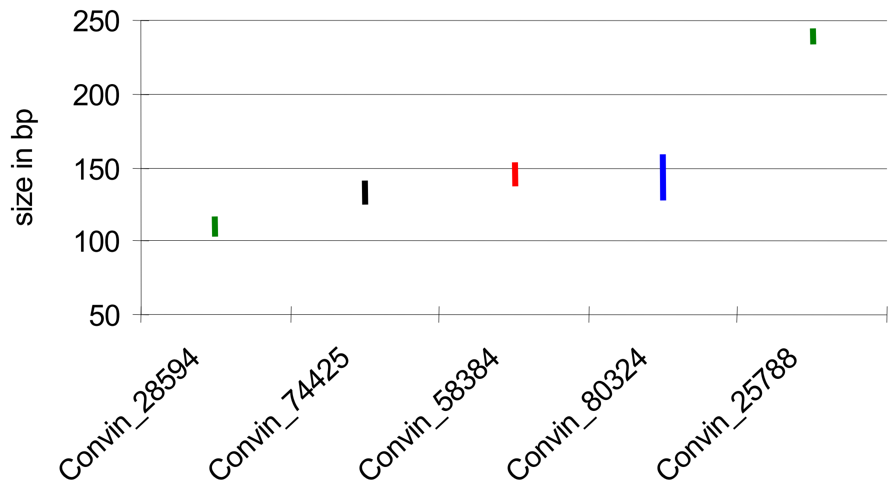

PCR Protocol Multiplex 3

Multiplex PCR amplification was optimized to be performed in a 10 µl reaction volume containing 5 µl HotstarTaq master mix (Qiagen, Cat. No 203445), double distilled water, and 0.3 µM of forward and reverse primers each (Table 5). We used the following thermotreatment on a TC-412 Programmable Thermal Controller (Techne): 35 cycles with 94°C for 30 seconds, 56°C for 90 seconds, and 72°C for 60 seconds. Before the first cycle, a prolonged denaturation step (95°C for 15 min) was included and the last cycle was followed by a 30 min extension at 72°C.

**Component**	**Primer mix concentration**	**Amount [µl]**	**Final amount/ concentration**
ddH_2O	-	1.2	1.2 µl
Qiagen 2x HotstarTaq Mastermix §	-	5	1 x
Convin_73025 (Atto550)	20 µM	0.3	0.3 µM each
Convin_70939 (Atto532)	20 µM	0.3	0.3 µM each
Convin_53403 (FAM)	20 µM	0.3	0.3 µM each
Convin_64790 (Atto565)	20 µM	0.3	0.3 µM each
Convin_72549 (Atto550)	20 µM	0.3	0.3 µM each
DNA	-	2	-

§ Provides a final concentration of 0.5 units HotStarTaq DNA polymerase, 1x PCR buffer with 1.5 mM $MgCl_2$ and 200 µM of each dNTP.

Fragment length distribution Multiplex 3

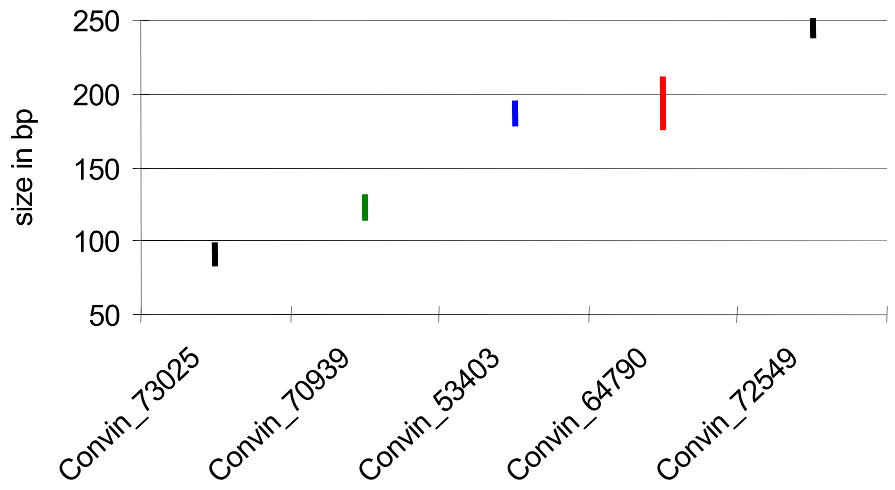

Dilution of primers in Multiplex 1 prior to PCR amplification

Locus	labelled F-Primer	unlabelled F-Primer	unlabelled R-Primer	ddH_2O	Final Concentration
Convin_02370	0.5 µl	4.5 µl	5 µl	40 µl	10 µM total F<br>10 µM total R
Convin_01805	0.5 µl	4.5 µl	5 µl	40 µl	10 µM total F<br>10 µM total R
Convin_08122	0.5 µl	4.5 µl	5 µl	40 µl	10 µM total F<br>10 µM total R
Convin_49091	0.5 µl	4.5 µl	5 µl	40 µl	10 µM total F<br>10 µM total R
Convin_63351	0.5 µl	4.5 µl	5 µl	40 µl	10 µM total F<br>10 µM total R

Dilution of primers in Multiplex 2 prior to PCR amplification

Locus	labelled F-Primer	unlabelled F-Primer	unlabelled R-Primer	ddH_2O	Final Concentration
Convin_28594	0.5 µl	4.5 µl	5 µl	40 µl	10 µM total F<br>10 µM total R
Convin_74425	0.5 µl	4.5 µl	5 µl	40 µl	10 µM total F<br>10 µM total R
Convin_58384	0.5 µl	4.5 µl	5 µl	40 µl	10 µM total F<br>10 µM total R
Convin_80324	2.5 µl	2.5 µl	5 µl	40 µl	10 µM total F<br>10 µM total R
Convin_25788	0.5 µl	4.5 µl	5 µl	40 µl	10 µM total F<br>10 µM total R

Dilution of primers in Multiplex 3 prior to PCR amplification

Locus	labelled F-Primer	unlabelled F-Primer	unlabelled R-Primer	ddH_2O	Final Concentration
Convin_73025	0.5 µl	4.5 µl	5 µl	40 µl	10 µM total F<br>10 µM total R
Convin_70939	0.5 µl	4.5 µl	5 µl	40 µl	10 µM total F<br>10 µM total R
Convin_53403	0.5 µl	4.5 µl	5 µl	40 µl	10 µM total F<br>10 µM total R
Convin_64790	0.5 µl	4.5 µl	5 µl	40 µl	10 µM total F<br>10 µM total R
Convin_72549	0.5 µl	4.5 µl	5 µl	40 µl	10 µM total F<br>10 µM total R

Table S1. Summary of significant linkage disequilibria among pairs of 15 microsatellite loci. The figures represent number of times a pair showed sign of linkage

locus	1	2	3	4	5	6	7	8	9	10	11	12	13	14
2	3													
3	1	0												
4	4	4	1											
5	4	3	0	3										
6	0	1	5	2	2									
7	2	0	2	2	4	1								
8	6	2	3	1	7	3	4							
9	2	0	4	9	3	2	2	4						
10	3	4	2	3	3	1	3	5	2					
11	7	2	2	1	6	2	3	5	2	0				
12	1	1	3	2	0	2	3	1	4	3	4			
13	2	1	3	2	0	2	1	3	4	5	3	9		
14	4	2	2	4	2	2	3	3	1	4	5	5	5	
15	0	1	2	4	2	1	3	3	3	3	3	5	4	6

Table S2. Significant departures from the Hardy-Weinberg equilibrium by Chi-squared test in study populations

population	locus	p-value
Ham	11	<0.001
Tul3	7	<0.001
	10	<0.001
	14	<0.001
Tul42	10	<0.001
	14	<0.001
Get	10	<0.001
	14	<0.001
Ver	2	<0.001
	5	<0.001
	8	<0.001
	10	<0.001
	11	<0.001
	15	<0.001
Em	11	<0.001
	14	<0.001
Res	11	<0.001
Lis	2	<0.001
	5	<0.001
	8	<0.001
	14	<0.001

Table S3. Observed frequency of null alleles across loci determined by two methods (Chakraborty et al. 1992, Brookfield et al. 1996) in raw data and adjusted proportions of null alleles estimated by the full model calculating inbreeding coefficient in the INEst software

locus	Chakraborty et al. (1992)	Brookfield et al. (1996)	INEst
1	0.33	0.27	0.01
2	0.62	0.29	0.08
3	0.17	0.13	0.03
4	0.24	0.21	0.08
5	0.41	0.33	0.07
6	0.29	0.24	0.10
7	0.49	0.37	0.06
8	0.36	0.31	0.02
9	0.19	0.17	0.06
10	0.43	0.39	0.03
11	0.53	0.40	0.02
12	0.18	0.14	0.10
13	0.23	0.18	0.03
14	0.50	0.34	0.04
15	0.32	0.23	0.12

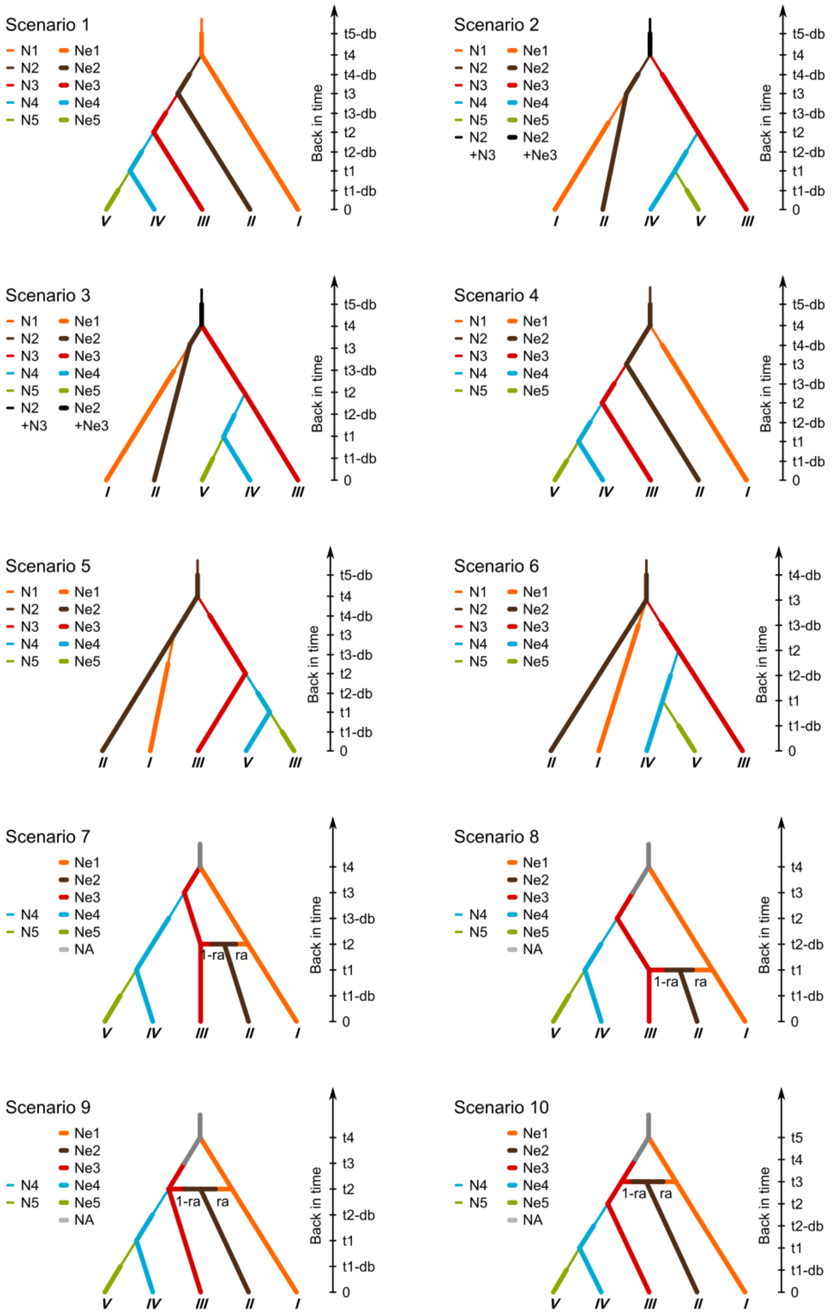

Fig. S1. Schematic representation of the 10 competing scenarios considered for the inference of the colonization routes of *C. vincitoxici* in Fennoscandia tested by the Approximate Bayesian Computation analysis. Five populations were considered in each analysis as determined by the clustering Structure analysis (Fig. 3). Parameters with associated prior distributions are described in Methods.

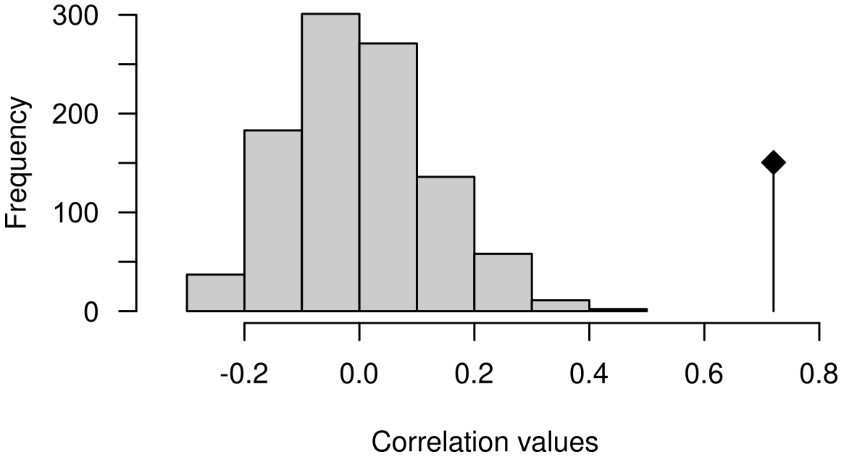

Fig. S2. Histogram of 999 permuted values under the absence of spatial structure and the original value of the Mantel correlation between genetic and geographic distance matrices (diamond).

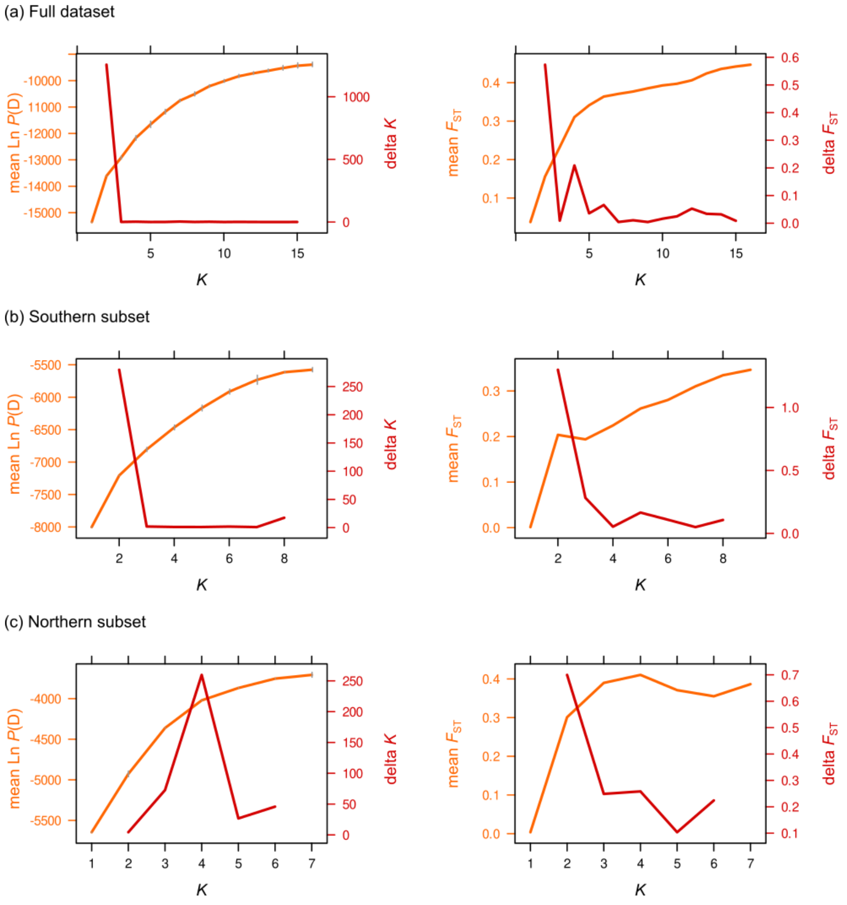

Fig. S3. Mean posterior probability (Ln P) and mean differentiation (F_{ST}) according to the number of simulated clusters (K) in an admixture model of Structure analysis for the full dataset and different subsets of populations. Vertical bars indicate ±SD.

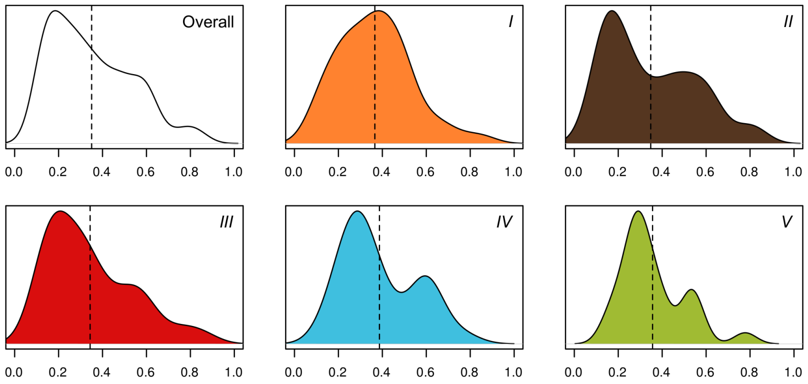

Fig. S4. Density plots of the inbreeding coefficient in all populations and inferred genetic clusters (I–V). Vertical dashed lines show mean values.
